# Supplementary material for: Development of a non-invasive diagnostic method for pathogenic RNA viruses using sebum wiped from the cat’s body surface
Source: Sci Rep. 2026 Feb 18;16:4101. doi: 10.1038/s41598-026-37655-z (PMC12916942; doi:10.1038/s41598-026-37655-z)
Supplement: Supplementary file 1 — Supplementary Material 1 [file 41598_2026_37655_MOESM1_ESM.pdf]

Supplementary Table 1. Ct value average of Fcwf-4 RNA and CRFK RNA

	Actb	***Cyc1***	***Gapdh***	***PPIA***	***Sdha***	***B2M***
**Ct value**	35.2	22.3	29.0	19.2	20.9	19.4

Supplementary Table 2. Sequences of primers/probe to amplify cat housekeeping genes

Target gene		Sequence (5'->3')	Tm	Length	Amplicon size
Actb	**Forward**	CGGCGCCGCCCTATAAA	59.9	17	144
	**Reverse**	TCATCATCCATGGCGAACTGAG	60.5	22	
Cyc1	**Forward**	TTGGAGTATGACGATGGCACC	60.1	21	149
	**Reverse**	AGAGGCAATAGCAAGCCCAT	59.5	20	
Gapdh	**Forward**	TGAAGGTCGGTGTGAACGG	59.9	19	134
	**Reverse**	CTGGAACATGTAGACCATGTAGT	57.7	23	
PPIA	**Forward**	GGTCAACCCCATCGTGTTTTT	59.3	21	79
	**Reverse**	GTCTGCAAACAGGTCGAAGG	59.1	20	
Sdha	**Forward**	CACTGACTAGGGCGCAGTGG	62.8	20	140
	**Reverse**	AAATTCATGGTCCACTACCGGG	60.4	22	
B2M	**Forward**	GCGTTTTGTGGTCTTGGTCC	63.7	20	96
	**Reverse**	GGGTGACGGGAGTAAACCTG	63.9	20	
	**Probe**	/5HEX/TGGATGCCG/ZEN/TCCAGCATTCTCCA/3IABkFQ/	69.7	23	

Supplementary Table 3. Sequences of primers and probe to amplify FIV

	Sequence (5’->3’)	Tm	Length	Amplicon size
**Forward**	GCCTTCTCTGCAAATTTAACACCT	63.9	24	91
**Reverse**	GATCATATTCTGCTGTCAATTGCTTT	62.7	26	
**Probe**	/5Cy5/CATGGCCAC/TAO/ATTAATAATGGCCGCA/3IAbRQSp/	67.7	25	

Supplementary Table 4. Sequences of primers and probe to amplify SFTSV

	Sequence (5'->3')	Tm	Length	Amplicon size
**Forward**	TGTCAGAGTGGTCCAGGATT	62.6	20	137
**Reverse**	ACCTGTCTCCTTCAGCTTCT	62.6	20	
**Probe**	/56-FAM/TGGAGTTTG/ZEN/GTGAGCAGCAGC/3IABkFQ/	67.1	21	
